# Clustered Associations between Musculoskeletal Fitness Tests and Functional Movement Screen in Physically Active Men

**DOI:** 10.1155/2023/5942329

**Published:** 2023-02-13

**Authors:** Jarosław Domaradzki, Dawid Koźlenia

**Affiliations:** Department of Biostructure, Wroclaw University of Health and Sport Sciences, Al. I.J. Paderewskiego 35, 51-612 Wrocław, Poland

## Abstract

Functional Movement Screen (FMS) is a tool used to assess fundamental movement patterns. There are relationships between musculoskeletal fitness (MSF) and a FMS. However, there is limited data regarding the multidimensional associations between these two constructs. This study is aimed at investigating the associations and detailed structures of FMS components and MSF components and identifying a deeper detailed structure of their relations to physical fitness. The study sample included 114 physically active men with an average body height of 1.81 ± 0.07 (cm), body weight of 80.61 ± 9.49 (kg), and a BMI of 24.65 ± 2.46 (kg/m^2^). Each participant performed a FMS test, sit-and-reach (S&R) test, sit-ups (ABS) evaluation, balance evaluation on an AMTI platform, handgrip strength (HG) testing, and standing broad jump (SBJ) ability. The Kendall's Tau correlation was performed to identify simple associations between FMS and MSF. Cluster analysis was used to determine the multidimensional nature of the relationships. In the vast majority, a positive correlation was observed suggesting that a high level of MSF improved FMS test results. A cluster analysis revealed 4 separate clusters. Shoulder mobility (SM) was identified as a single cluster. A strong similarity was observed between SBJ and trunk stability push-up (TSPU) forming a second cluster. This cluster joined to another consisting of the S&R test and active straight leg raise (ASLR) test. The last cluster consisted of HG and hurdle step (HS). These results confirmed that FMS and MSF tests measure the same constructs—a foundation for an individual's motor coordination, muscle strength, postural stability, and dynamic balance. This knowledge could be helpful in effectively enhancing physical performance based on combining similar constructs to accelerate the achievement of established goals.

## 1. Introduction

Being physically active is a critical component of a healthy lifestyle. Numerous studies have documented the health benefit and effects on well-being associated with a physically active lifestyle [1, 2]. Participation in sports, such as sports competitions or recreational activities, requires a high level of physical fitness, motor skills, and quality movement. The common biological foundation consists of muscular strength, motor coordination, and neuromuscular control [3]. All these areas should be integrated. Low-quality movement patterns may negatively affect musculoskeletal performance [4] and lead to an increased risk of injury [5].

A Functional Movement Screen (FMS) is a battery of tests developed by Cook et al. [6, 7]. It is a valuable tool for assessing fundamental movement performance in mobility, stability, neuromuscular coordination, and functional asymmetry. Deficiencies or low-quality fundamental patterns are viewed as a risk factor for physical activity-related injuries [8, 9]. However, some observations deny the usefulness of FMS in injury risk prediction [10, 11], whereas others have postulated the meaning of other related factors that influence the reliability of the FMS [12, 13]. Therefore, FMS scores may be useful but should be interpreted with caution [14]. Some observation did not provide results that confirmed low quality movement efficiency in injury risk prediction [10, 11]. The FMS is used in medicine and physiotherapy to evaluate the quality of movement but can also be used to assess one's individual performance in sports or physical activities [6, 7]. Every FMS module and overall test scores can reveal movement dysfunctions to predict the risk of musculoskeletal injuries [15]. The test has been validated many times, and its reliability has been positively confirmed [16, 17].

In a health-related fitness (H-RF) concept, muscular strength, endurance, flexibility, and postural balance are built together from a separate construct—musculoskeletal fitness (MSF) [18, 19]. MSF is considered a health marker [20]. Each item of MSF can predict one's health status in childhood, adolescence, and later phases of life [21]. Thus, the musculoskeletal system's efficiency can be considered one of the conditions of physical health.

Due to similar biological (anatomy-physiological) foundations, fundamental movement patterns and MSF are related [21, 22]. The constructs measured using the FMS and MSF tests are indicative of an individual's ability in motor coordination, muscle performance, range of motion (ROM) of the joints, and balance control of body posture [22].

Many studies assessing the associations between FMS and MSF tests have confirmed this relationship [3, 8, 9, 15, 23, 24]. Positive correlations have been found between flexibility and agility tests and FMS scores [4, 23]. Flexibility seems to be correlated to FMS scores [25, 26]. Additionally, lower limb power and trunk muscle strength were related to the quality of movement patterns [27–29]. Also it was showed the relationship between movement patterns quality and speed abilities [30]. But, on other hand, a few observations failed to find a correlation between FMS scores and 10-meter sprints, 20-meter sprints, and vertical jumps [31, 32]. But, on the other hand, there were more observations identifying a relationship between FMS and MSF, as well as between MSF and the quality of movement patterns [4, 9]. Therefore, exploration of these associations is needed. Most studies evaluated competitors participating in sports, particularly team sports [15, 23, 26, 29]. There is a lack of studies on the average physically active population [33]. Moreover, the most frequently examined features were strength, speed, and agility [31, 32]. To date, there is limited research regarding the relationship between FMS and flexibility, balance, and strength [9], and in most analyses, simple correlations (Spearman or Pearson) were used [31, 32]. According to our knowledge, no studies have assessed the multidimensional relationship between FMS and MSF tests using cluster analysis. Thus, our study aimed to investigate the associations and detailed links between FMS modules and basic MSF tests and to identify clusters of the most similar items. The cluster analysis gave a new insight into a multidimensional database containing sets of FMS and MSF tests.

## 2. Material and Methods

### 2.1. Participants

Our study included 114 physically active male volunteers with a mean age of 22.98 ± 1.68. The selection of a group of young, physically active men resulted from the assumption that physical activity would provide an appropriate level of motor skills and movement patterns, which would differentiate more than in the group of physically inactive people. The Senate Research Ethics Committee at the Wroclaw University of Health and Sport Sciences approved the study. The ethical requirements for our human experiments were in accordance with the Helsinki Declaration (consent number 16/2018). The research was conducted in the Biokinetics Research Laboratory at the Wroclaw University of Health and Sport Sciences, which has a Quality Management System Certificate of PN-EN ISO 9001: 2009 (Certificate Reg. No. PW-48606-10E). Individuals were required to sign a written informed consent form before participating in the study. Each person was informed in detail about the aim of the study, the type of study, the research methodology, and the participation conditions. All were familiarized with the examination procedures and the requirements of both physical and technical tests. Participants were allowed to withdraw from the research at any time without giving a reason.

Exclusion criteria included a current injury that limited sports participation, an injury occurring 6 weeks prior to the start of the study, and no involvement in sports or physical activities in the last 5 years.

### 2.2. Measurements

The measurements performed were associated with those described in our previous research [9].

Body height and weight were measured, and the participant's body mass indexes (BMIs) were calculated. A SECA model 764 anthropometer was used (SECA manufactured, Hamburg, Germany. Quality control number C-2070). To determine the quality of movement patterns, a FMS was conducted. Additionally, the MSF test was performed (Functional Movement Systems, Inc., Chatham, USA). The FMS consisted of 7 single movement tasks [6, 7]. All of them were awarded 3 points when the observed movement was performed correctly, 2 when visible compensation was identified, 1 when it was impossible to perform the movement, and 0 when pain was experienced while performing the movement. The tasks evaluated included deep squat (DS), trunk stability push-up (TSPU), and bilateral tests such as the hurdle step (HS), in-line lunge (IN-L), shoulder mobility (SM), active straight leg raise (ASLR), and rotary stability (RS) were carried out on the left and right side with the lower grade between sides being used for analysis. The maximum score possible on the FMS test is 21 points. A score of 14 points or less was associated with low-quality movement patterns and a growing association with the risk of injury [8].

For leg power, the standing broad jump (SBJ) was conducted. The examined subjects stood in front of a designated line, jumped, and landed on both legs while swinging the upper limbs. The measurement from the line to the heels was conducted.

The sit-and-reach (S&R) test was performed to examine lower back and hamstrings flexibility. To conduct this test, a table and measuring tape were needed. The examined subject sat with straight legs, and their feet were placed on the sidewall of the table. They then bent at the torso as far as possible with their legs straight and moved the measuring tape on the tabletop.

The S&R test measures flexibility and was conducted using a table with the following dimensions: 35 cm long, 45 cm wide, and 32 cm high with a tabletop of 55 cm long. The measuring tape was positioned parallel to the long axis of the table, measuring zero to 50 cm. The tabletop protruded 15 cm above the sidewall against which the subject placed their feet. On the tabletop, an indicator was loosely applied and was moved by the participant with his hands to obtain the measurements. The subjects sat down with straight legs and both feet positioned flat against the table's sidewall. The participant moved the indicator across the measuring tape as far as they could while maintaining straight legs.

Handgrip strength (HG) was used to evaluate the strength of the upper limb. This test used a hydraulic dynamometer with an adjustable grip, SAEHAN SH5001 (manufacturer: Saehan Corporation, South Korea).

The sit-ups test was conducted to evaluate abdominal muscle strength. This test consists of bending the torso from a lying position and touching their knees with their elbow. Scoring was based on how many they could perform in 30 seconds.

Balance was evaluated using the ACCU SWAY stabilometric platform (Advanced Mechanical Technology, Inc. (AMTI), Newton, MA, USA). The examined subject stood for 30 seconds without shoes and maintained a standing position. The analyzed parameters were the distance traveled from the examined subject's center of gravity and the field's perimeter (COP Area).

### 2.3. Statistical Analysis

There were two sets of variables evaluated in our study: continuous (anthropometric measurements, motor test results, and FMS overall results) and ordinal (FMS components). The normality of the distribution of continuous variables was tested using the Shapiro-Wilk test. Descriptive statistics were calculated and presented with the mean ± standard deviations for continuous variables and medians and interquartile ranges for ordinal variables.

We used cluster analysis—a multidimensional method to evaluate the interrelationship between variables within a data set. Usually, the set of variables in cluster analysis is consistent (variables measured on the same scale, e.g., continuous variables). In these situations, a simple standardization approach is sufficient to fulfill the assumptions required of cluster analysis. In cases of mixed data, the traditional approach is unable to meet these assumptions. However, there are several methods to fix this problem. In our study, we adopted the approach presented in economic sciences (where continuous variables, e.g., family income, are often agglomerated with ordinal variables such as social-economic variables, e.g., the number of children in a family) [34, 35].

One assumption of cluster analysis is to select the correct agglomeration distance (adequate to the data set). This assumption was met in our study by selecting the generalized distance measurements (GDM) algorithm developed by Walesiak [36, 37]. GDM is based on Kendall's Tau (*τ*) correlation matrix. Kendall's *τ* correlations are used when the assumption of normality cannot be met (or there are different variables in the data set: continuous and ordinal) [38]. The structure of Kendall's *τ* correlation is valid to implement in mathematical operations using an ordinal scale. In addition, inspecting simple correlations at the start of the analysis made it possible to evaluate the correlations between variables and fulfill the subsequent assumption that the data should not be strongly correlated.

Cluster analysis agglomerates variables and groups with the most similar features inside the clusters and those that differ outside the cluster. In comparison to simple correlations, this method gives deeper insight into the structure of associations between both sets of tests.

Grouping was done using Ward's method [39]. This procedure provides a structure that develops groups with the minimal variance within the clusters and maximum variation between the clusters. The graphical result is dendrogram groups of variables that differ the least and are connected on a given level of similarity. The dendrogram is a representation of the internal structure of the associations between measured features. Mojena criterion (upper tail rule) was used to select the optimal grouping of results obtained from the agglomerative cluster analysis method (hierarchical). The method is based on an analysis of fusion levels of objects in the dendrogram to determine their cut-off point, i.e., to choose the optimal clustering result [40].

## 3. Results

Descriptive characteristics of the anthropometric measures, MSF test results, and FMS overall scores are shown in Table 1.

Descriptive statistics of the different components of the FMS test are presented in Table 2.

In the first stage of assessing the associations between MSF and FMS, Kendall's *τ* correlation coefficients were investigated. Results of the correlations are presented in Table 3.

The relationships were positive, which suggests that a high level of MSF supported FMS test results. An especially strong association was observed between SBJ and ASLR, IN-L, and DS. S&R was linked to DS and ASLR. The COP Area was related only to the HS.

**Figure 1:**
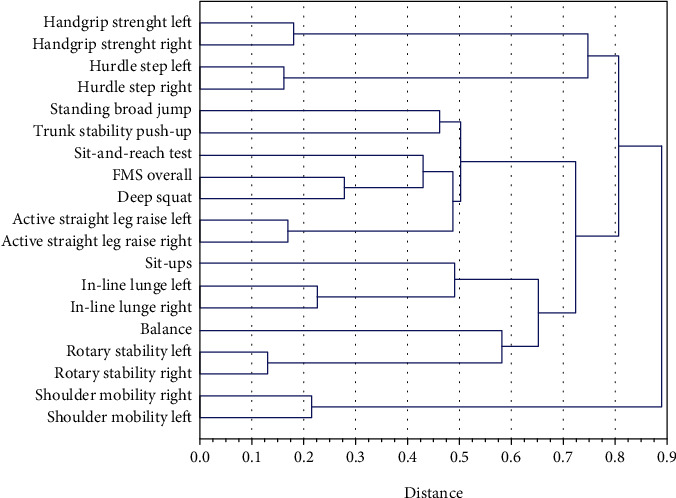
Cluster analysis of the MSF and FMS components.

The second stage of assessing the associations between MSF and FMS tests was to investigate the clusters of variables. Dendrograms illustrated the hierarchical structure of the analyzed variables based on the decreasing similarity of these traits (Figure 1).

We observed four agglomerations with small clusters inside each. The agglomerative coefficients did not exceed 0.9. HS (left and right) constituted a separate cluster that included HS (left and right) in the group. Standing broad jumps and trunk stability push-ups were linked to another massive cluster formed by the S&R test, FMS overall, DS, and ASLR (left and right). The measure of suit-ups was linked to in-line lunges (left and right) and loosely (agglomerative coefficient: 0.64) to postural balance, which was lined to RS (left and right). SM (left and right) formed a single separate cluster.

## 4. Discussion

This study is aimed at assessing the intercorrelations between MSF and FMS components. These intercorrelations are manifested in groups of variables agglomerated with cluster analysis to show clustered associations. The presented approach to the mentioned problem allowed for a deeper insight into the hierarchical structure of functional traits and the abilities of the MSF and FMS. Specifically, we performed a detailed exploration of the links between strength, muscle power, flexibility, postural balance, and functional movement patterns.

Hierarchical cluster analysis is an exploratory method identifying structure within the data. It is often used to identify homogenous groups of cases. Similar participants are agglomerated in clusters, while there is a dissimilarity between participants across clusters [34]. Thus, cluster analysis results allow distinctively separate individuals with different levels of motor performance or/and movement patterns. Additionally, the agglomeration of variables revealed connections between motor skills and specific movement pattern components. It indicated that individuals included in different clusters differed in their motor levels and movement patterns. It allows for indirect conclusions about the presented movement patterns based on motor skills and distinguishes individuals.

HG correlated with the HS. The relationship between the left hand and HS was significant. The reason for a lack of a simple correlation between the right hand and HS (right and left) is unclear. In cluster analysis, both hand measurements were very closely connected to both HS assessments. FMS overall correlated significantly with the S&R test, and both were agglomerated close together suggesting a strong connection between the factors observed in other studies [23, 25, 26]. FMS overall was agglomerated with the long jump and the distance achieved [29]. The ASLR was also present in this agglomeration (likewise a significant correlation was present), suggesting a strong relationship between the abovementioned factors [41]. Deep squats were similarly agglomerated with the FMS overall. Deep squats were also significantly correlated with S&R and SBJ. In-line lunges (both sides) were clustered together with the sit-ups test. However, we did not find similar associations. Despite SM correlating significantly with HS (in simple Kendal's *τ* relationships), these factors were separated in cluster analysis by a long distance. Although RS and trunk stability push-ups were related to balance, there was no simple correlations with balance test results [42].

Our study sample presents, on average, high-quality movement patterns (FMS > 14) in agreement with other observations among groups of physically active subjects. A similar observation was noted in physically active groups like footballers [43], runners [44], volleyballers [45], and the other athlete population [46].

Analyses of the relationship between the quality of movement patterns and MSF showed a statistically significant correlation between flexibility (S&R scores), FMS overall, ASLR, and DS. Positive correlations were also observed when evaluating FMS overall, DS, ASLR, and SBJ. Similar observations were noted in other studies, especially regarding the relationships between flexibility and the quality of movement patterns. Glass et al. [47] showed associations between a higher level of strength, balance, and flexibility with FMS scores. Grygorowicz et al. [26] observed a better-quality of movement pattern with a higher level of flexibility. Jenkins et al. [25] observed that hip ROM relationships are associated with a higher quality of movement patterns. The observations confirmed by Chimera et al. [41] showed that worse ROM of the hip and knee joints negatively affects the quality of movement patterns. Moreover, these authors [41] also showed that stronger trunk muscles positively influenced the FMS. In our research, ABS was associated with the IN-L test, which confirms the role of abdominal muscle stabilization in this movement. Bagherian et al. [48] using postural muscle training noticed a clear improvement in the quality of movement patterns in the research group after an 8-week training intervention. In the context of lower limb power, Sannicandro et al. [49] found that if footballers had better quality movement patterns, the greater their lower limb power. Willigenburg and Hewett [29] observed correlations between SBJ and overall FMS scores among American football players. In our own analysis, HG showed an association with MS. However, Silva et al. [28] did not observe a relationship between HG and the quality of movement patterns. Similarly, in terms of balance, the results are mixed. We observed HG relationships in our subjects, but Atalay et al. [42] did not observe any relationships.

Cluster analysis reveals a less homogeneous structure and more segmentation. Both spheres of MSF and FMS are intertwined with close links. The relationships between individual MSF and FMS components are more clearly identified.

The first aggregation was the most extensive. It contained 7 elements grouped into 3 clusters. It included two trials on MSF and 5 on FMS. SBJ was associated with TSPU. Power ability is represented by SBJ, which engages core stability when arm swing is utilized, which allows force transfer between the upper and lower body. The relationships between these features were not strong as confirmed by the agglomeration coefficient. S&R was associated with hip mobility and pelvic stabilization (performed in the DS trial). The flexibility test requires the flexibility of muscle groups of the deep back, hamstrings, abdominal strength, and iliolumbar muscles. DS attempts to assess the quality of movement patterns that depends on the control of muscle tension in the back and lower limbs, including the hamstrings muscles. The same neuromuscular mechanism is used in activities requiring active mobility in the flexion and hyperextension of the hips [50, 51]. Such actions are illustrated by the ASLR test which is closely related to the previous tests.

The second agglomeration is comprised of 6 elements. It related the IN-L trials, which require the control and stabilization of the torso and pelvis, to the deep lunge that requires stabilization on one leg. The strength of the torso muscles (ABS, especially the abdomen) is essential for activities that require body stabilization [52]. On the other hand, control of an equivalent posture is determined by activities involving muscular stabilization while balancing on both legs and the conditions of instability of the support performed in the RS test (in FMS) [53]. Therefore, the connection of the COP area with RS is the result of their common neurophysiological basis, i.e., complex neuromuscular coordination, torso strength, and torso stabilization [54].

The third agglomeration included static strength (local upper limbs (HG) and stability one leg stance), core strength and mobility, and stability of pelvis screening through HS. This association may not be apparent. It is difficult to explain using a common structural or functional basis [55]. They relate to different parts of the body. Perhaps, the relationship results from the method of performing both tests which require stabilization of the shoulder and upper limbs while the subject holds the crossbar over their head.

The last, fourth agglomeration included two SM trials. This test is based on ROM and muscle control [56]. This activity is very independent of other abilities included in both tests, MSF and FMS, which is confirmed by a high agglomeration coefficient (0.8).

There are some limitations concerning our study. Considering cluster analysis with a large set of variables, it seemed more participants should be examined (second shortcoming) using a narrower range of age such as focusing only on 22 years old adults.

## 5. Conclusions

This research is aimed at investigating the clustered associations between MSF components and the FMS test. Cluster analysis was used to develop a detailed structure of links between several functional measurements. The functional structure was segmented (4 clusters). Knowledge of the exact multidimensional relationships between movement patterns and muscle strength and power, flexibility, and postural balance can be helpful for physiotherapists and sports staff when introducing adequate training programs (based on MSF components) to improve the quality of movement patterns and to compensate imbalances in strength, mobility, and stability. Also, functional asymmetries could be eliminated using adequate exercises. On the other hand, FMS is suitable for determining motor abilities with tests that could be used to stimulate and develop motor strength, power, flexibility, and coordination. This study confirmed the FMS and MSF tests measured similar constructs including the foundation for an individual's motor coordination, muscle strength, postural stability, and dynamic balance.

## Figures and Tables

**Table 1:** Descriptive statistics of anthropometric measurements, BMI, MFS tests, and FMS overall.

Variable	Mean (±sd) 95% CI
Body height (cm)	1.81 (0.07)
	1.8-1.82
Body weight (kg)	80.61 (9.49)
	78.85-82.37
BMI (kg/m^2^)	24.65 (2.46)
	24.2-25.11
Left hand grip (kg)	54.83 (8.19)
	53.31-56.35
Right hand grip (kg)	56.7 (8.52)
	55.12-58.28
Standing broad jump (cm)	216.54 (28.91)
	211.15-221.93
Sit-ups (*n*)	30.48 (4.77)
	29.6-31.37
Sit-and-reach (cm)	16.86 (9.68)
	15.07-18.66
COP area (cm^2^)	2.44 (1.74)
	2.12-2.77
FMS overall (pts)	14.43 (3.12)
	13.85-15.01

**Table 2:** Descriptive statistics of component tests of FMS.

Variable	Me (±IQR) 95% CI
Deep squat	2 (1)
	1.74-2.04
Hurdle step left	2 (1)
	2.06-2.29
Hurdle step right	2 (1)
	2.13-2.35
In-line lunge left	3 (1)
	2.33-2.57
In-line lunge right	3 (1)
	2.21-2.49
Shoulder mobility left	2 (1)
	1.99-2.29
Shoulder mobility right	3 (1)
	2.28-2.55
Active straight leg raise left	2 (1)
	2.1-2.37
Active straight leg raise right	2 (1)
	2.1-2.37
Trunk stability push-up	2 (0)
	2.49-2.72
Rotary stability left	2 (0)
	1.9-2.06
Rotary stability right	2 (0)
	1.88-2.04

**Table 3:** Kendall's Tau correlation between FMS components and MSF test.

Test	HG left	HG right	SBJ	ABS	S&R	COP area
FMS overall	0.05	0.04	0.14^∗^	0.05	0.15^∗^	0.00
Deep squat	0.00	0.02	0.20^∗^	0.04	0.28^∗^	0.05
Hurdle step left	0.02	0.04	0.05	0.03	-0.10	-0.13^∗^
Hurdle step right	0.14^∗^	0.11	0.07	0.05	-0.06	-0.06
In-line lunge left	0.05	0.07	0.22^∗^	0.10	0.07	-0.06
In-line lunge right	0.00	-0.03	0.14^∗^	0.19^∗^	-0.15^∗^	0.03
Shoulder mobility left	-0.22^∗^	-0.16^∗^	-0.01	-0.17^∗^	0.10	-0.02
Shoulder mobility right	-0.11	-0.04	0.01	-0.10	0.07	0.03
Active straight leg raise left	0.14^∗^	0.16^∗^	0.18^∗^	0.01	0.22^∗^	-0.10
Active straight leg raise right	0.13^∗^	0.09	0.25^∗^	0.00	0.22^∗^	-0.02
Trunk stability push-up	0.12	0.09	0.07	0.04	-0.02	0.05
Rotary stability left	0.02	-0.08	0.14^∗^	0.09	-0.09	0.06
Rotary stability right	-0.06	-0.13^∗^	0.10	0.03	-0.02	0.07

Abbreviations: HG L: handgrip left; HG R: handgrip right; SBJ: standing broad jump; ABS: suit-ups test; S&R: sit-and-reach test; COP Area; a center of gravity area.

## Data Availability

The data presented in this study are available upon request from the corresponding author.
